# Oral amiodarone and propranolol in maintenance therapy of postimplantation tachycardia: An observational study

**DOI:** 10.1097/MD.0000000000038839

**Published:** 2024-07-12

**Authors:** Abdulla Arslan, Öykü Gülmez Özkaya, Fatih Aytemiz, Hakan Altay, Emin Evren Özcan, Deniz Süha Küçükaksu, Vedat Bakuy, Ömer Kozan, Seçkin Pehlivanoğlu

**Affiliations:** a Cardiology Department, Baskent University Istanbul Medical and Research Center, Altunizade/Istanbul, Turkey; b Cardiology Department, Manisa State Hospital, Manisa, Turkey; c Cardiology Department, Dokuz Eylül University Izmir Medical and Research Center, Balçova/Izmir, Turkey; d Cardiovascular Surgery Department, Baskent University Istanbul Medical and Research Center, Altunizade/Istanbul, Turkey.

**Keywords:** left ventricular assist device, left ventricular tachycardia, oral amiodarone, oral propranolol

## Abstract

Left ventricular assist devices (LVADs) are widely used as end-stage therapy in patients with advanced heart failure, whereas implantation increases the risks of development of sustained ventricular tachycardia at the later postimplantation stage. Therefore, this study aimed to evaluate the clinical efficacy of orally administered amiodarone and propranolol in 3 patients with ventricular tachycardia (VT) after LVAD implantation who were resistant to initial anti-antiarrhythmic drugs. This retrospective cohort study consisted of the initial evaluation of the clinical data of 14 adult patients who underwent implantation of LVAD between January 2019 and March 2021. A total of 3 patients with resistant VT were finally included. In all cases, the patients were initially administered amiodarone in the different doses intravenously to stabilize the critical condition, whereas its oral form along with that of propranolol was used as maintenance therapy in the first 2 cases. In the third case, amiodarone was withdrawn because of the risk of development of hyperthyroidism, while oral propranolol was used in the treatment. The assessment in the 16-month follow-up period after discharge did not show presence of non-sustained and sustained VT in all 3 cases. In the ventricular arrhythmia-free group, the total mortality rate within the follow-up period was 11.1 ± 7.78 months in the 3 patients. We suggest that maintenance oral therapy of propranolol and amiodarone can significantly decrease the risks of complications in patients with VT after implantation of ventricular assist device in the long term.

## 1. Introduction

Currently, left ventricular assist device (LVAD) implantation is considered the most effective treatment for end-stage advanced heart failure (AHF).^[1]^ LVAD implantation is increasingly used in patients with AHF as a bridge therapy to cardiac transplantation or end-stage treatment.^[2]^

**Figure 1.**
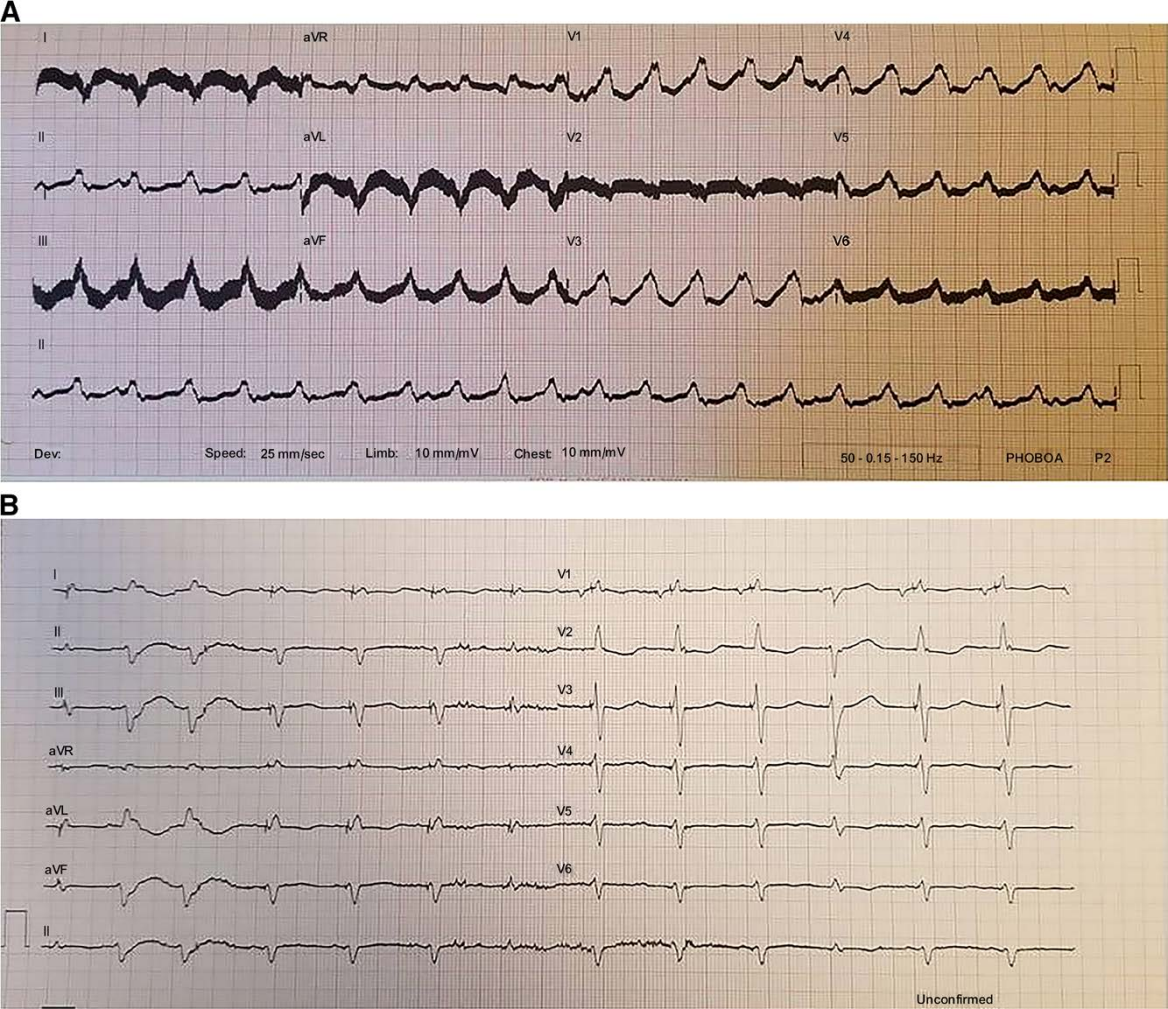
Continuous flow-left ventricular assist devices shown at the surgical implantation (A) and the model (B).

Continuous flow (CF)-LVADs are the devices wherein blood can be drawn via the cannula located in the apical region of the left ventricle (LV) and is transmitted to the aorta with the help of a constant-speed rotor (Fig. 1). In contrast to pulsatile flow, the CF physiology causes high sympathetic activity^[3]^ and difficult-to-control hypertension, while it increases alpha1-receptor-mediated total peripheral resistance.^[4]^ Furthermore, the high shear of plasma products and blood passing through the rotor and propeller of the CF-LVAD creates stress, which can cause acquired von Willebrand syndrome and increased bleeding. Anticoagulant use further complicates the situation.^[5,6]^

Therefore, patients with LVAD implantation are already at risk of ventricular tachycardia (VT) due to AHF. Sustained ventricular tachycardia (SVT) (>30 seconds of ventricular ectopic beats (VESs) and/or hemodynamically unstable) occurs in 22% to 47% patients 1 year after LVAD implantation.^[7]^ VT occurrence increases thrombosis and stroke risk in its turn. Thus, VT in patients with LVAD implantation affects their mortality by causing secondary conditions rather than inducing arrhythmias that directly cause sudden death.^[8,9]^ Nearly half of all patients with LVAD implantation have at least one life-threatening VT event within the first year after implantation. Some studies showed that VT occurring early after LVAD implantation decreased the overall survival rate.^[10]^ Although guidelines suggest that selective beta-blockers plus amiodarone can be administered as antiarrhythmic therapy in the early postoperative phase, some studies showed that combined oral propranolol and amiodarone therapy was superior in patients with recurrent VT.^[11]^ However, no uniform consensus was achieved, as the data on antiarrhythmic therapies in patients with LVAD implantation are controversial. A particular study by Refaat et al^[12]^ found the strong association between the nonuse of beta-blockers and incidence of VA. On the contrary, more recent studies demonstrated that these medications could not produce the desired effect, as their dosage had to be reduced because of the adverse effect on right ventricular function.^[13]^

Hence, this study aimed to evaluate the clinical efficacy of oral amiodarone and propranolol in 3 patients with SVT after LVAD implantation as maintenance therapy taking into consideration the underlying pathophysiological mechanisms.

## 2. Methods

### 2.1. Ethical statement

The study protocol was in accordance with the Declaration of Helsinki and was approved by the Baskent University Institutional Review Board (Approval No. KA21/159). The requirement for informed consent was waived because of the retrospective study design.

We retrospectively reviewed the arrhythmic data of pre-LVAD and post-LVAD implantation in 14 adult patients who underwent LVAD implantation between January 2019 and March 2021. The exclusion criteria were the postinfarction condition, severe aortic insufficiency and stenosis, pulmonary hypertension, end-stage renal disease, and the presence of malignant tumors. The measurement procedures included a review of the patients’ daily medical and telemetry records and implantable cardioverter-defibrillator (ICD) interrogations for the documented preoperative and postoperative evidence of non-sustained VT (NSVT) and sustained VT (SVT). Clinically significant ventricular arrhythmia (VA) was defined as ventricular fibrillation (VF), SVT, or NSVT with symptoms requiring antiarrhythmic therapy. NSVT was defined as ≥3 consecutive ventricular ectopic beats lasting <30 seconds at a rate of >100 beats/minute (bpm), whereas SVT was considered as that having >30 seconds of ventricular ectopic beats with a rate > 100 bpm. Furthermore, in all patients with ICD, arrhythmia density was assessed upon the investigation of the ICD interrogations. Patients without postoperative evidence of VT or NSVT in their daily medical records, 24-hours daily telemetry records, and ICD interrogations were considered the VA-free cohort after LVAD implantation. Finally, the 3 cases of SVT were included in the study for representation, where the relevant antiarrhythmic therapy was performed.

### 2.2. Follow-up period in the studied groups

There was a 16-month follow-up period after discharge for both groups, where the recurrence of SVT and NSVT and mortality rate along with the complications were assessed in the study and control groups.

## 3. Results

### 3.1. Cases with SVT after LVAD implantation

**Figure 2.**
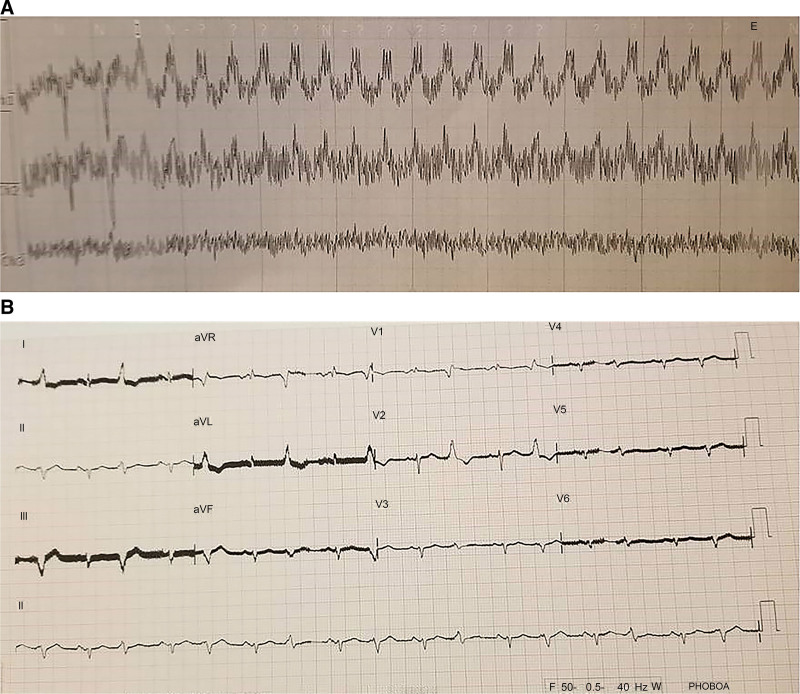
Electrocardiography (ECG) during ventricular tachycardia (A) versus basal ECG (B) ECG. The obviously longer pattern for QT segment is suggestive of SVT.

Case 1 involved a 60-year-old man with coronary artery disease (CAD), paroxysmal atrial fibrillation hypertension, and diabetes mellitus. Although the patient was under optimal medical therapy for ischemic heart failure (HF) (carvedilol 12.5 mg/day, sacubitril/valsartan 200 mg/day, ivabradine 15 mg/day, atorvastatin 20 mg/day, apixaban 10 mg/day, and cardiac resynchronization therapy defibrillator, CRT-D; Dynagen™ X4, Boston, MA), he was classified under the class 4 status using the New York Heart Association (NYHA) and Interagency Registry for Mechanically Assisted Circulatory Support (INTERMACS) classifications. Thus, LVAD implantation was selected as a bridge therapy to cardiac transplantation. Transthoracic echocardiography (TTE) data, right heart catheter findings, and antiarrhythmic therapy data are presented in Table 1. A HeartWare™ ventricular assist system (Medtronic Inc., Minneapolis, MN) was successfully implanted. Within 24 hours after the procedure, SVT was observed (Fig. 2). The CRT-D device did not apply shocks, as it was only triggered when the heart rate exceeded 160 bpm. Intravenous (IV) amiodarone (150 mg) was administered, and considering the ongoing arrhythmia, 100 mg lidocaine was also used, yielding a sinus rhythm. For maintenance therapy, 200 mg/day oral amiodarone was added, and carvedilol was switched to 80 mg/day propranolol. Apixaban therapy was replaced with warfarin sodium. During hospitalization and at the 16-month follow-up after discharge, SVT and NSVT did not recur.

**Table 1:** Transthoracic echocardiography and catheterization findings.

	Case 1	Case 2	Case 3
LVEF %	19	15	13
LV diameter	58 × 66 mm	56 × 60 mm	75 × 82 mm
TAPSE	18 mm	15 mm	12 mm
PA pressure	56/22/35 mm Hg	52/24/36 mm Hg	72/31/44 mm Hg
PCWB	32 mm Hg	31 mm Hg	34 mm Hg
RV pressure	50/0 to 8 mm Hg	48/0 to 7 mm Hg	69/0 to 13 mm Hg
RA pressure	16 mm Hg	13 mm Hg	10 mm Hg
ECG rhythm	A sense, biventricular pace	Sinus rhythm	Sinus rhythm
Preoperative antiarrhythmic therapy	Carvedilol 12.5 mg/d	Metoprolol 100 mg/d, amiodarone 100 mg/d	Bisoprolol 5 mg/d, amiodarone 400 mg/d
Postoperative antiarrhythmic therapy	Amiodarone 200 mg/d, propranolol 80 mg/d	Amiodarone 100 mg/d, propranolol 160 mg/d	Mexiletin 400 mg/d, propranolol 120 mg/d

ECG = electrocardiography, LV = left ventricle, LVEF = left ventricular ejection fraction, PA = pulmonary artery, PCWB = postcapillary wedge pressure, RA = right atrium, RV = right ventricle, TAPSE = tricuspid annular plane systolic excursion.

**Figure 3.**
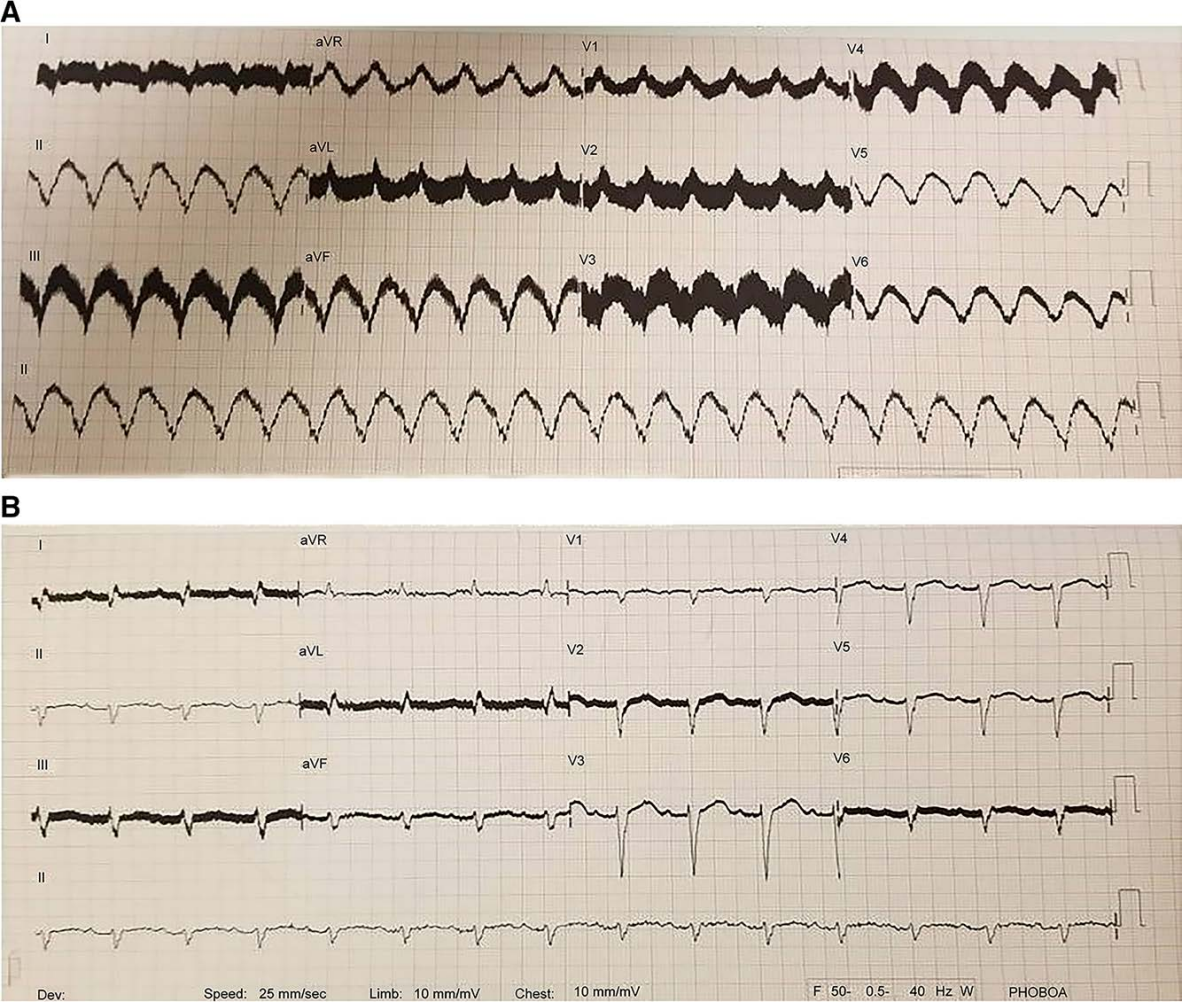
Ventricular tachycardia monitored in a rhythm Holter (A) versus basal ECG (B) 1 day after the procedure. ECG = electrocardiography.

Case 2 involved a 52-year-old woman with CAD and recurrent VT. The patient was followed for ischemic HF and had a Visia AF single chamber (VVI; Medtronic Inc.) ICD. As she was classified under NYHA 4 and INTERMACS 4 and hospitalized thrice in the past 6 months with recurrent decompensated HF attacks, LVAD implantation was selected. TTE and right heart catheter findings are presented in Table 1. SVT was not observed in the previous month’s ICD records of the patient under rivaroxaban (20 mg/day), amiodarone (100 mg/day), metoprolol (100 mg/day), ramipril (5 mg/day), and spironolactone (50 mg/day). A HeartMate 3 (Abbott, Chicago, IL) was successfully implanted. VT with a tachycardia cycle length (TCL) of 382 ms was observed after 32 hours (Fig. 3). No hemodynamic deterioration was observed during invasive arterial blood pressure monitoring. The VVI-ICD did not provide shock because it was set to trigger at heart rates exceeding 174 bpm. As VT persisted after IV amiodarone 300 mg, IV lidocaine 100 mg was added, and sinus rhythm was achieved. At the follow-up, metoprolol was switched to 160 mg/day oral propranolol and 200 mg/day oral amiodarone. Rivaroxaban therapy was replaced with warfarin sodium. During the in-hospital follow-up, VT did not recur. As the QT interval on electrocardiography (ECG) was 510 ms after the amiodarone dosage was increased, amiodarone was initially discontinued. After 2 days, it was resumed at a lower dosage (100 mg/day) when the QT interval was 460 ms. During the 16-month follow-up, no SVT and NSVT were observed.

**Figure 4.**
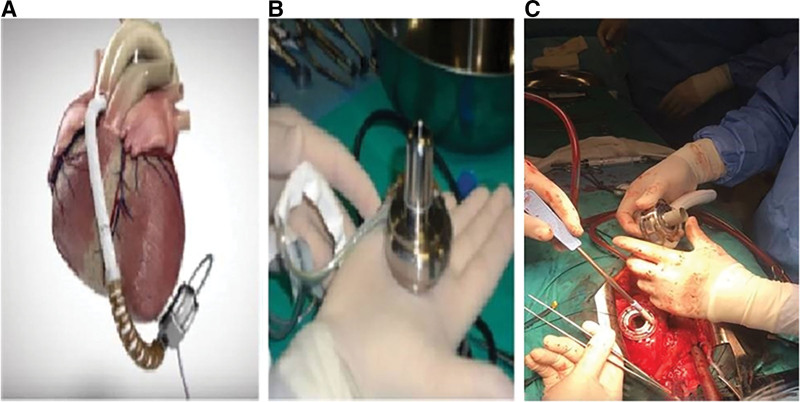
Electrocardiography (ECG) during ventricular tachycardia (A) versus basal ECG (B, C) ECG in case 3.

Case 3 involved a 67-year-old man with CAD, peripheral arterial disease, and ischemic HF. A PERCIVA™ dual chamber (DDD; Boston Scientific, Boston, MA) ICD was implanted as secondary prophylaxis owing to the patient’s history of SVT. He received 5 mg/day bisoprolol, 400 mg/day amiodarone, 200 mg/day sacubitril and valsartan, and 25 mg/day spironolactone. There was no VT in the previous 3 months’ ICD records. After 3 hospitalizations for decompensated HF in the past 3 months, he was classified under NYHA 4 and INTERMACS 4 and hospitalized for LVAD implantation. TTE and right heart catheterization were performed (Table 1). The heart team selected LVAD implantation as a bridge therapy to heart transplantation, and a HeartMate-3 (Abbott, USA) was successfully implanted. SVT was observed after 96 hours (Fig. 4). The DDD-ICD device did not apply shock, as it was set to trigger when the heart rate exceeded 168 bpm. While the patient’s hemodynamics was closely monitored, a sinus rhythm was not achieved with an IV bolus of 300 mg amiodarone. Thus, 50 mg IV lidocaine and 40 mg/h IV amiodarone were infused, along with 2 mg IV magnesium. However, VT continued and an ICD controller device was requested. When the patient’s hemodynamics deteriorated before the device was supplied, the LVAD pump flow rate decreased from 3.5 to 2.4 L/min. Thus, IV fluid replacement was initiated, and the patient was defibrillated (150 J) under sedation using an external defibrillator with anterior–posterior patches. Thereafter, a sinus rhythm and improved hemodynamics were observed. For maintenance, 120 mg/day oral propranolol was administered, and amiodarone was replaced with 400 mg/day mexiletine owing to the development of amiodarone-induced hyperthyroidism. The patient was discharged on day 9 after the procedure. At the 15-month follow-up, no SVT and NSVT were noted.

At the 15-month follow-up after discharge, no SVT and NSVT were observed in the ICD records of all patients. Moreover, in the 24-hours rhythm Holter recordings, the VES burden was significantly decreased at the 1-month follow-up.

### 3.2. VA-free cases after LVAD implantation

Eleven patients (9 men, 2 women; mean age, 58.6 years) had LVAD and were assessed for VAs. The mean left ventricular ejection fraction was 20.18 ± 3.31 (range, 13–26; median, 20), and the INTERMACS level of all patients were 4 or 5. A total of 14 patients received LVAD as destination therapy; 3 of these patients received it as a bridge to destination therapy. Five patients had a HeartMate-3, another 5 had a HeartWare™ system, and 1 had a HeartMate-2. The detailed medication treatment is described in Table 2. Nine patients were followed for an average of 11.1 ± 7.78 months (median, 9 months; range, 4–26 months). The total mortality rate was recorded for 3 patients. In-hospital mortality included 3 patients: 1 died a day after LVAD implantation because of low right heart output, while the other died because of arrhythmia due to uncontrolled infection on the 12th day. Another patient died 5 months after implantation because of pump thrombosis and major bleeding during the treatment (Table 2).

**Table 2:** Patients’ demographics and perioperative and postoperative characteristics.

Patient no.	Age (yrs)	Date of implantation	Mortality date	Follow-up period (months)	LVAD type	History of arrhythmia	Etiology of heart failure	LVEF	RV function
1	54	February 11, 2019	–	26	HeartMate-3	None	Ischemic	20	Mild–moderately depressed
2	53	July 7, 2019	–	21	HeartMate-3	None	Nonischemic	18	Moderately depressed
3	63	September 31, 2019	February 24, 2020	5	HeartMate-2	None	Ischemic	26	Moderately depressed
4	57	November 4, 2019	November 16, 2019	12 d^a^	Heart Mate-3	Positive	Nonischemic	24	Moderately depressed
5	50	February 3, 2020	–	14	BVAD-HeartWare	None	Nonischemic	20	Severely depressed
6	55	July 20, 2020	–	9	HeartWare	None	Nonischemic	20	Normal function
7	60	July 23, 2020	–	9	HeartWare	None	Ischemic	20	Mildly depressed
8	61	August 27, 2020	–	8	Heart Mate-3	Positive	Ischemic	20	Mildly depressed
9	72	December 21, 2020	–	4	HeartWare	None	Ischemic	13	Mild–moderately depressed
10	65	December 23, 2020	–	4	HeartWare	None	Nonischemic	22	Normal function
11	56	January 18, 2021	January 19, 2021	1 d^a^	Heart Mate-3	Positive	Nonischemic	19	Moderate–severely depressed

LVAD = left ventricular assist device, LVEF = left ventricular ejection fraction.

a This follow-up period only includes the in-hospital period because of mortality.

## 4. Discussion

LVAD implantation in patients with HF can be associated with postoperative risks, one of which is supposed to be VT, and it can be explained by several possible mechanisms. The application of oral propranolol and amiodarone in clinical practice can alleviate the existing symptoms and eliminate the risks of further complications.

First, cannula-related VTs were observed in >50% of patients in the first month after LVAD implantation. In addition to chronic ventricular scars, de novo scars at LVAD entry cannulation sites provided potential substrates for reentry arrhythmia. Thus, VT originating from the lateral wall of the left ventricle (LV) in the event of non-myocarditis, from the LV septum due to sarcoidosis, and from the apical aneurysm-induced scar tissue in the event of anterior myocardial infarction can occur, and not all VTs were caused by de novo scar tissue. Three-quarters (75%) of paired VTs in patients with ablation were associated with intrinsic myocardial scars instead of entrance cannula scars.^[14]^ De novo scar-related VT associated with the implanted cannula was considered to have a right bundle branch block (RBBB) morphology, left superior axis, and precordial transition between V3 and V5. The ECG in case 3 had a RBBB morphology, left superior axis, and precordial passage in V3. The TCL was 380 ms. The apical LV septum region, possibly the LVAD apical cannula circumference, was considered the VT origin. Early and late VTs were caused by scar tissue far from the inflow cannula at varying frequencies.^[9,11]^ Scar-related reentry VTs caused significant morbidity and mortality in patients with AHF. However, in these patients, it was also thought that the myocardium around the cannula was alive, thus creating less substrate for VT.^[9]^

As the autonomic nervous system has an important impact on arrhythmogenesis, the decrease in baroreflex sensitivity owing to decreased catecholamine levels and degraded vagal reflexes play important roles in arrhythmia occurrence.^[15,16]^ In patients with HF, the sympathetic tone increases to provide adequate contraction. Particularly, a positive inotropic response and cell growth are related to the 3 main adrenergic receptors (beta 1, beta 2, and alpha 1). While the beta 1/beta 2 receptor rate in healthy hearts is 70/30 in LV and 80/20 in the right ventricle, it is 60/40 in an insufficient heart owing to beta 1 receptor downregulation. Thus, selective beta 1 receptor blockade can facilitate continuous sympathetic signal transmission through unblocked cardiac beta 2 receptors that are not cardiostimulatory but can increase VA risk. In contrast, beta 2 receptor blockade may be useful, considering that beta 2 receptors are not downregulated in HF.^[17]^ Postinfarction studies also suggest that nonselective blocking agents can provide more protection against catecholamine toxicity than those acting on beta 1 receptors.^[18]^ Moreover, propranolol is liposoluble and thus has the potential to block central and prejunctional receptors in the central nervous system, independent of its effect on peripheral beta receptors, thereby creating a local anesthetic effect by blocking sodium channel conduction.^[19]^ The impact of sympathetic blockade during treatment has been identified, and the association of propranolol with amiodarone has proved to be very effective in patients with ICD.^[11]^

A constant antiarrhythmic agent was not used in our patients. Antiarrhythmic therapy was started with propranolol in our first patient; in our second patient, the beta-blocker therapy along with the antiarrhythmic therapy that he had already received was switched, and the antiarrhythmic therapy in our last patient was replaced by an alternative 3 days after propranolol initiation. Thus, propranolol is recommended as the first-choice beta-blocker regardless of the antiarrhythmic preference in patients with VT after LVAD implantation.

Second, in the acute and chronic periods after LVAD, the left ventricular end-diastolic pressure began to decrease, leading to elevated remodeling in the extracellular matrix. As shown in the retrospective study by Harding et al,^[21]^ the corrected QT (QTc) was prolonged in the first week after LVAD implantation and subsequently decreased. Prolonged QT increased VT events 3-fold.^[20]^ Thus, the trigger activity, which was caused by repolarization abnormality, in addition to the conventional physical reentry and automaticity, may cause another new mechanism for VT events after LVAD implantation. However, the observed decrease in the QTc time and QRS interval after the 1-week period and prolonged time of LVAD mechanical support created positive electrophysiological remodeling, which explained the decreased frequency of VT over time. Furthermore, patients with chronic HF could also have prolonged myocyte action potential, which could lead to prolonged QTc and electrophysiological abnormalities with increased heart rate variability.

In this way, propranolol proved to demonstrate its effects through beta-adrenergic and non-beta-adrenergic receptors. Similar to other beta-blockers, it shortens the QT interval by reducing the action potential duration. It also significantly shortened the monophasic action potential duration in electrophysiological studies in dogs, resulting in an increased ratio of the ventricular effective refractory period to the monophasic action potential time and shortened QT interval. These effects may not have been mediated by beta-adrenergic receptors.^[22]^ It is not conclusively proven that VT in patients with LVAD implantation is adrenergically mediated. Therefore, this study aimed to determine the influence of propranolol on VT suppression and daily VES burden by administering the highest tolerable dose, which can be easily achieved owing to the systemic circulation provided by LVAD. In that study, the metoprolol-administered group had a 77.5% lower inhibition of arrhythmia than the propranolol-administered group. Propranolol was associated with a 2.67-times decrease in the VT or VF event rate per patient in a 48-hours period. A significant effect of propranolol in preventing arrhythmias was observed from the 7th hour post, which was attributed to a more effective sympathetic blockade.^[9]^ When our patients were switched to propranolol as a beta-blocker, along with newly added or continued amiodarone use, the number of monitored VES markedly decreased in the first 24 hours. After propranolol initiation, no patient had VT. In case 3, mexiletine replacedamiodarone because of amiodarone toxicity. In case 2, amiodarone was discontinued as the QTc was 510 ms on treatment day 2; amiodarone was resumed at 100 mg/day after 2 days when the QT interval was 460 ms. We planned to increase the propranolol dose in patients according to the decrease in VES burden and VT occurrence, but the desired responses were obtained at doses of 80 to 160 and 120 mg/day, respectively.

In some studies, the most important risk factors for VT events after LVAD implantation were described as VT history, atrial fibrillation rhythm, and history of cardiac surgery.^[18]^ There are some contradictions in the description of VT risk after LVAD implantation among studies. While some of them found a higher risk in patients with ischemic HF,^[23,24]^ others determined the same for patients with nonischemic HF.^[25]^ Although the absence of postoperative beta-blocker use, long basal QTc, and electrolyte imbalance are considered risk factors for VT, none have been fully confirmed.^[8,26,27]^ A history of VT was the strongest predictor for VF after LVAD implantation.^[28,29]^

There are no medical treatment recommendations for VA after LVAD implantation. Beta-blockers are generally accepted to be useful, but there is no evidence for the optimal beta-blocker. We treated our patients in the light of studies conducted in patients with HF. Propranolol is thought to be particularly effective in atrial arrhythmia and VA caused by catecholamine or digoxin toxicity; however, generally, it is effective in controlling recurrent VT and VES. However, its effects are dose-dependent, and tolerance can potentially limit high-dose administration for effective arrhythmia control. Given that no previous studies have systematically examined this issue, we obtained this information from a few case reports and small-scale studies.^[27]^ In small-scale studies in patients with AHF who underwent ICD implantation, propranolol plus amiodarone was more effective, safer, and superior than metoprolol plus amiodarone.^[9]^

The limitations of the study are the small sample size and retrospective design. No uniform therapy with oral amiodarone and propranolol was applied, as in some cases it had to be discontinued. Similarly, the patients had the different stages of HF, therefore a broader study having greater sample size and enabling to perform the comparison between the different types of groups is required.

We conclude that propranolol is well-tolerated by patients with LVAD implantation and can be administered in higher doses. Thus, propranolol is the preferred beta-blocker for this patient group. This medication can be used in high doses in patients with LVAD implantation, act on the central nervous system receptors through its liposoluble characteristics, and provide significantly reduced catecholamine discharge by nonselective beta-blockade. Thus, we propose that propranolol can make a significant difference in this cohort and may be considered a first-choice beta-blocker. Similarly, the combination therapy with amiodarone proved to have the compatible clinical efficacy. Larger cohort studies are needed to verify our results and determine the preferable pharmacological agents that may effectively reduce VA rates in patients with LVAD.

## Acknowledgments

We would like to thank Enago (www.enago.com) for the English language editing.

## Author contributions

**Conceptualization:** Abdulla Arslan, Öykü Gülmez Özkaya, Fatih Aytemiz.

**Data curation:** Abdulla Arslan, Öykü Gülmez Özkaya, Fatih Aytemiz, Hakan Altay, Emin Evren Özcan, Deniz Süha Küçükaksu, Vedat Bakuy.

**Writing – original draft:** Abdulla Arslan, Öykü Gülmez Özkaya, Fatih Aytemiz.

**Writing – review & editing:** Abdulla Arslan, Öykü Gülmez Özkaya, Fatih Aytemiz, Hakan Altay, Emin Evren Özcan, Deniz Süha Küçükaksu, Vedat Bakuy, Ömer Kozan, Seçkin Pehlivanoğlu.

**Formal analysis:** Ömer Kozan, Seçkin Pehlivanoğlu.

**Conceptualization:** Abdulla Arslan, Öykü Gülmez Özkaya, Fatih Aytemiz.

**Data curation:** Abdulla Arslan, Öykü Gülmez Özkaya, Fatih Aytemiz, Hakan Altay, Emin Evren Özcan, Deniz Süha Küçükaksu, Vedat Bakuy.

**Writing – original draft:** Abdulla Arslan, Öykü Gülmez Özkaya, Fatih Aytemiz.

**Writing – review & editing:** Abdulla Arslan, Öykü Gülmez Özkaya, Fatih Aytemiz, Hakan Altay, Emin Evren Özcan, Deniz Süha Küçükaksu, Vedat Bakuy, Ömer Kozan, Seçkin Pehlivanoğlu.

**Formal analysis:** Ömer Kozan, Seçkin Pehlivanoğlu.
